# Supplementary material for: Mechanisms governing the pioneering and redistribution capabilities of the non-classical pioneer PU.1
Source: Nat Commun. 2020 Jan 21;11:402. doi: 10.1038/s41467-019-13960-2 (PMC6972792; doi:10.1038/s41467-019-13960-2)
Supplement: Supplementary file 7 — Source data [file 41467_2019_13960_MOESM7_ESM.zip › Source_Data/Figure6/Figure6B_MotifScanOutput/ETS1_mutPU.1/homerResults/motif30.similar.html]

motif30

## Information for motif30

T
A
G
C
G
T
A
C
T
G
A
C
T
G
C
A
A
T
G
C
G
T
C
A
T
G
C
A
C
G
T
A
A
T
G
C
G
A
T
C
C
T
A
G
  
Reverse Opposite:  

G
A
T
C
C
T
A
G
T
A
C
G
G
C
A
T
A
C
G
T
C
A
G
T
T
A
C
G
A
C
G
T
A
C
T
G
C
A
T
G
A
T
C
G
  

|  |  |
| --- | --- |
| p-value: | 1e-66 |
| log p-value: | -1.540e+02 |
| Information Content per bp: | 1.525 |
| Number of Target Sequences with motif | 499.0 |
| Percentage of Target Sequences with motif | 3.70% |
| Number of Background Sequences with motif | 409.8 |
| Percentage of Background Sequences with motif | 1.17% |
| Average Position of motif in Targets | 103.0 +/- 53.5bp |
| Average Position of motif in Background | 103.4 +/- 63.8bp |
| Strand Bias (log2 ratio + to - strand density) | -0.1 |
| Multiplicity (# of sites on avg that occur together) | 1.01 |
| Motif File: | file (matrix) reverse opposite |

### Similar de novo motifs found

|  |  |  |  |  |  |  |  |
| --- | --- | --- | --- | --- | --- | --- | --- |
| Rank | Match Score | Redundant Motif | P-value | log P-value | % of Targets | % of Background | Motif file |
| 1 | 0.848 | A G C T T A G C G A T C A G T C G T C A A T G C G T C A T C G A C T G A T A G C G T A C G C T A | 1e-59 | -136.185779 | 4.69% | 1.89% | motif file (matrix) |
